# Pelvic splenosis—A rare cause of pelvic mass

**DOI:** 10.1002/ccr3.2419

**Published:** 2019-09-16

**Authors:** Jad A. Degheili, Nassib F. Abou Heidar

**Affiliations:** ^1^ Division of Urology Department of Surgery American University of Beirut‐Medical Center Beirut Lebanon

**Keywords:** pelvis, spleen, splenosis, Technitium‐99m sulfur colloid scintigraphy

## Abstract

Splenosis is a medical condition that seldom occurs after splenic tissue spillage via trauma or surgery. Ectopic spleen tissues can be found almost anywhere within the body. Albeit benign, it is often misdiagnosed as a tumor. Surgery is not indicated unless symptomatic. The imaging of choice for pelvic splenosis, although conventional, is a sulfur colloid nuclear scintigraphy.

## Graphical abstract

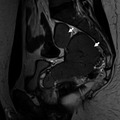

## 1 CASE

**Figure 1:**
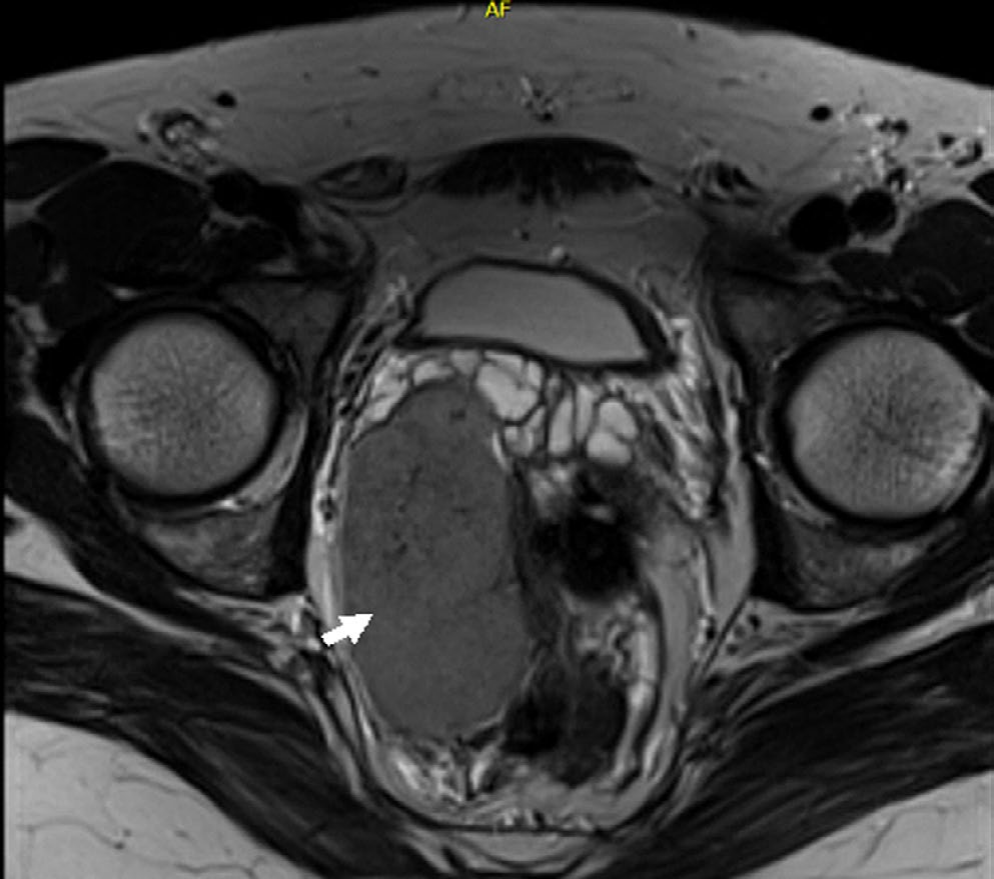
Axial view of a magnetic resonance imaging (MRI) of the pelvis, T2‐weighted sequence, showing a large pelvic well‐defined mass measuring around 12 by 10 cm, causing compression on the right seminal vesicle, and causing mild compression and deviation on the rectum without definite invasion

**Figure 2:**
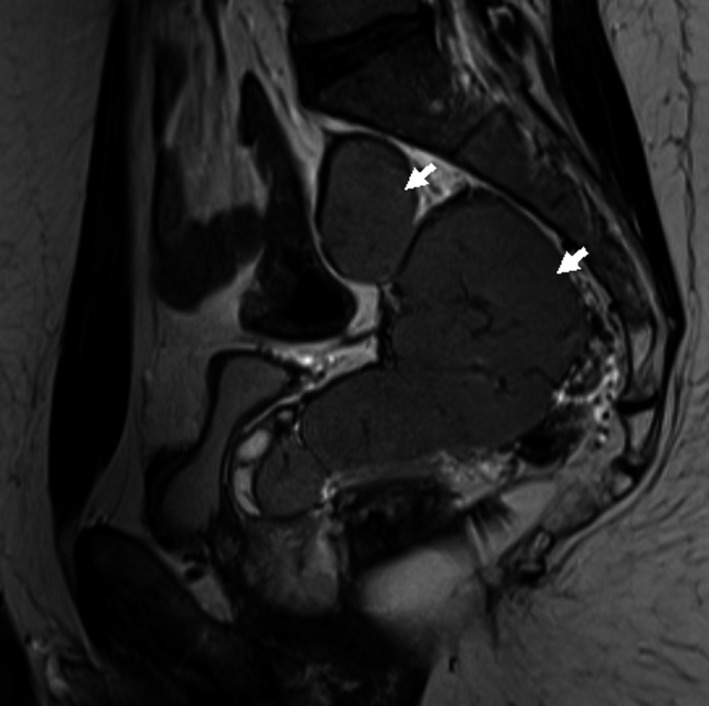
Sagittal view of a magnetic resonance imaging (MRI) of the pelvis, T2‐weighted sequence, showing the same huge pelvis mass consisting of two adjacent well‐defined masses in the right perirectal space, the larger measuring 10 × 8 cm, compressing the right seminal vesicle and rectum

**Figure 3:**
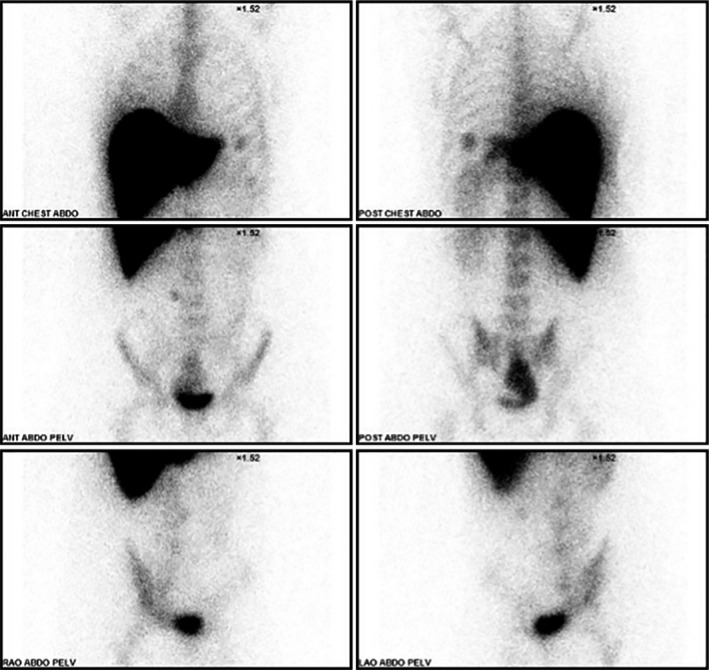
Technitium‐99m sulfur colloid scintigram showing increased uptake of the pelvic mass consistent with a large pelvic spleen (arrow), along with smaller other nodules in the subdiaphragmatic area consistent with small scattered splenules (arrowhead)

A 34‐year‐old male patient with prior history of post‐traumatic splenectomy in his early childhood presented for investigation of his infertility. Semen analysis showed severe oligospermia, but otherwise normal testosterone and follicular stimulating hormone levels. Pelvic ultrasound performed for vague nonspecific flank pain revealed an incidental large pelvic mass. Further imaging with a multiparametric pelvic MRI showed a 12 × 10 cm right pelvic mass compressing the right seminal vesicle and deviating the rectum laterally (Figures 1 and 2). The signal intensity and enhancing pattern were suggestive of a large pelvic splenosis. For better confirmation, a Technitium‐99m sulfur colloid scintigram was performed that established this index lesion to be of splenic origin (Figure 3; arrow), in addition to other smaller splenules (Figure 3; arrowhead). As no excision was performed, patient elected to proceed with in vitro fertilization. A 6‐month to 1‐year follow‐up ultrasound imaging was recommended for the pelvic spleen.

Splenosis is usually an incidental finding and commonly occurs after splenic trauma or after splenectomy, 1 and can present as an asymptomatic finding on imaging.^2^ It is thought to result from spilled splenic tissue that receive blood supply from surrounding vessels, unlike accessory spleens which derive their blood supply from the splenic artery.^1^ Malignant pelvic masses remain a paramount concern for physicians; however, Technitium‐99m sulfur colloid is the diagnostic test of choice that precludes a surgical procedure or biopsy.^1^ As such, splenosis must be on the differential for any abdominal/pelvic masses in postsplenectomy patients.

## CONFLICT OF INTEREST

None of the contributing authors has any conflict of interest, including specific financial interest or relationships and affiliations relevant to the subject matter or materials discussed in the manuscript.

## AUTHOR CONTRIBUTIONS

JD and NAH: wrote the manuscript, performed the literature review on the subject, and approved the final version of the manuscript prior to submission.

## CONSENT

Written informed consent was obtained from the patient for publication of this case and any accompanying images. A copy of the written consent is available for review by the Editor‐in‐Chief of this journal upon his request.
